# An integrated workplace mental health intervention in a policing context: Protocol for a cluster randomised control trial

**DOI:** 10.1186/s12888-016-0741-9

**Published:** 2016-02-27

**Authors:** Anthony D. LaMontagne, Allison J. Milner, Amanda F. Allisey, Kathryn M. Page, Nicola J. Reavley, Angela Martin, Irina Tchernitskaia, Andrew J. Noblet, Lauren J. Purnell, Katrina Witt, Tessa G. Keegel, Peter M. Smith

**Affiliations:** Centre for Population Health Research, School of Health & Social Development, Deakin University, Geelong, VIC 3220 Australia; Faculty of Business and Law, Deakin Business School, Deakin University, Geelong, 3220 VIC Australia; Melbourne School of Population and Global Health, University of Melbourne, Parkville, Melbourne, VIC Australia; Tasmanian School of Business and Economics, University of Tasmania, Sandy Bay Campus, Hobart, TAS Australia; College of Science, Health and Engineering, School of Psychology and Public Health, La Trobe University, Melbourne, VIC Australia; Monash Centre for Occupational & Environmental Health, School of Public Health & Preventive Medicine, Alfred Hospital, Monash University, Melbourne, VIC Australia

**Keywords:** Workplace mental health, Job stress, Stress reduction, Mental health literacy, Cluster RCT, Intervention

## Abstract

**Background:**

In this paper, we present the protocol for a cluster-randomised trial to evaluate the implementation and effectiveness of a workplace mental health intervention in the state-wide police department of the south-eastern Australian state of Victoria. n. The primary aims of the intervention are to improve psychosocial working conditions and mental health literacy, and secondarily to improve mental health and organisational outcomes.

**Methods/design:**

The intervention was designed collaboratively with Victoria Police based on a mixed methods pilot study, and combines multi-session leadership coaching for the senior officers within stations (e.g., Sergeants, Senior Sergeants) with tailored mental health literacy training for lower and upper ranks. Intervention effectiveness will be evaluated using a two-arm cluster-randomised trial design, with 12 police stations randomly assigned to the intervention and 12 to the non-intervention/usual care control condition. Data will be collected from all police members in each station (estimated at >20 per station). Psychosocial working conditions (e.g., supervisory support, job control, job demands), mental health literacy (e.g., knowledge, confidence in assisting someone who may have a mental health problem), and mental health will be assessed using validated measures. Organisational outcomes will include organisational depression disclosure norms, organisational cynicism, and station-level sickness absence rates. The trial will be conducted following CONSORT guidelines. Identifying data will not be collected in order to protect participant privacy and to optimise participation, hence changes in primary and secondary outcomes will be assessed using a two-sample t-test comparing summary measures by arm, with weighting by cluster size.

**Discussion:**

This intervention is novel in its integration of stressor-reduction and mental health literacy-enhancing strategies. Effectiveness will be rigorously evaluated, and if positive results are observed, the intervention will be adapted across Victoria Police (total employees ~16,500) as well as possibly in other policing contexts, both nationally and internationally.

**Trial registration:**

Current Controlled Trials: ISRCTN82041334. Registered 24th July, 2014.

## Background

Mental health problems account for roughly a quarter of total years lost due to disability and are the third largest cause of the overall disease burden in Australia [1, 2]. The majority of these mental health problems occur in working age Australians, with one in five Australians aged 16-85 years experiencing an anxiety, affective or substance use disorder in any given year [3]. Past studies across a large number of countries have demonstrated that stressful working conditions, such as the combination of high job demands and low job control (job strain), have detrimental impacts on mental health [4–6]. In Australia, previous research has estimated a job strain-population attributable risk (PAR) for depression of 13 % among working males and 17 % among working females [7], and an associated cost burden of $730 M per year nationally [8]. This is only a fraction of the total depression-related workplace costs which have been estimated at $12.6B per annum [8]; but this fraction likely underestimates the job stressor-attributable burden of mental health problems because other job stressors (e.g., bullying, job insecurity) and other mental health problems associated with job stressors (e.g., anxiety, burnout) have not been accounted for [8]. Job stressors also predict elevated risks for poor health behaviours as well as various physical illnesses, including cardiovascular disease [9, 10].

In parallel to the growing recognition of and responses to job stress, interventions to promote mental health and mental health literacy in the workplace are gaining acceptability as a means to prevent, screen, and effectively manage depression, anxiety, and other mental health problems among employees in various industrialised countries [11–15]. Mental health literacy is defined as knowledge and beliefs about mental disorders which aid their recognition, management or prevention [16]. A prominent Australian example of this is *beyondblue’s* National Workplace Program, which aims to raise awareness of depression and anxiety as treatable illnesses, improve help-seeking behaviours, reduce stigmatising attitudes, and develop confidence and skills in providing help to people who might be experiencing a mental illness [17]. Another example is Mental Health First Aid (MHFA), which seeks to improve mental health literacy by developing knowledge and skills on how to recognise common mental disorders and provide “First Aid” support until professional help can be obtained, increase understanding about the causes of mental disorders, improve knowledge of the most effective treatments, and reduce stigma [18, 19]. There is evidence of effectiveness of MHFA from various studies [19], including randomised-controlled trials [18] and cluster randomised controlled trials [20]. In addition to improvements in mental health literacy, there is also some evidence of improvements in mental health among MHFA trainees [18]. These programs address some aspects of mental health literacy, but to date they have tended to emphasise reducing the impact of mental health conditions or providing strategies to manage mental health conditions in the long term (secondary and tertiary prevention), rather than preventing mental health conditions from occurring in the first instance (primary prevention).

In the workplace setting, primary prevention should include reduction of work-related risks to mental health, as well as enhancement of mental health-promoting aspects of work [14]. Job stress prevention features prominently here, and is relevant in all work contexts [21]. Job stress interventions have tended to focus on the primary and secondary intervention levels, whereas mental health literacy interventions have tended to focus on the secondary and tertiary levels, and the two have tended to operate independently [10]. A more integrated approach would bring these together to encompass primary, secondary, and tertiary intervention.

There is growing recognition among workplace stakeholders of the need to fulfill Occupational Health and Safety (OH&S) obligations with respect to psychological and physical health, as well as growing awareness of the impact of common mental disorders (work-related or otherwise) on productivity at work (e.g., sickness absence, presenteesim, turnover) [8, 11, 14, 22, 23]. Employers, in particular, are increasingly receptive to integrated or comprehensive approaches, as characterised above, which to some extent are practiced in Europe [24] and Canada [25], but less so in Australia and elsewhere [10, 14]. Canada’s 2013 “National standard on psychological health and safety in the workplace” represents a recent policy development aiming to promote integrated approaches to workplace mental health using a voluntary standard strategy [25, 26].

While all occupational groups are potentially exposed to job stressors, some occupations are more exposed than others. Research in the Australian context [27, 28] as well as internationally [29] has identified police work as being particularly stressful. High levels of job stressors in police have been linked to burnout, work-family conflict [30], depression, intimate partner violence [31], psychological distress [27, 28], and suicide [32]. As in other occupations, organisational sources of job stress such as high job demands (e.g., time pressures and work overload), low supervisor or collegial support [27, 28, 33, 34] and low levels of control (i.e., latitude in deciding how to do one’s work) have been found to be significant sources of stress in police [27, 28, 33]. While stress-induced mental and physical health outcomes in law enforcement may also be linked to acute operational situations and events such as attending accident scenes and exposure to violence [35, 36], evidence to date suggests that organisational sources of job stress are better predictors of police distress [37–39] than acute and potentially traumatic incidents.

There have been a number of job stress intervention studies in the police sector [40, 41]. However, these have tended to focus on improving individual responses to stressors, rather than the reduction of stressors. Given that many of the stressors experienced by police stem from both individual and organisational sources, it is appropriate to address intervention efforts at both these levels. This is further supported by the findings of systematic reviews of job stress intervention studies, which indicate that the most effective interventions combine secondary worker-directed (e.g., coping and time management skills) with primary work-directed intervention (e.g., moderation of demands, improved supervisory support) [42, 43].

The current study will be undertaken in conjunction with three partner organisations, namely Victoria Police, WorkSafe Victoria and the Victorian Health Promotion Foundation (referred to as VicHealth). Victoria Police is committed to finding more effective ways of protecting and promoting the mental health of its members and will be hosting the trial. Victoria Police is one of the largest employers in Victoria (~16,500 employees), and has one of the highest job stress-related claims burdens in the workers compensation system. The second partner, Worksafe Victoria, is the OH&S regulator and state workers’ compensation insurance provider. Worksafe is keenly interested in preventing work-related psychological harm and reducing associated claims in Victoria Police. Worksafe funds this research through the Institute for Safety Compensation and Rehabilitation Research (ISCRR). Finally, the Victorian Health Promotion Foundation, the third project partner, has a long-standing interest in workplace health promotion, and in developing new intervention strategies in the workplace setting [44]. This trial was preceded by two intervention needs assessment and development projects in Victoria Police (described below), also involving the above-named partners.

In this paper, we present the protocol for a cluster-randomised trial to evaluate the implementation and effectiveness of a workplace mental health intervention in 24 Victoria Police stations. The following main intervention effectiveness hypotheses will be tested comparing the intervention versus the control arms:

1. There will be significantly greater improvement in the intervention compared to wait-list control arms in the primary outcomes of mental health literacy and psychosocial working conditions over a one year period;
2. There will be significantly greater improvement in the intervention versus control arms in the secondary outcomes of mental health and organisational outcomes over a one year period;

The intervention will be targeted, and hypotheses tested, at the police station (cluster) level. The randomisation procedure, outcome measurement, and analysis will also be conducted at the station or cluster level.

## Methods/design

### Design of the study

A two-arm cluster RCT will be used to evaluate intervention effectiveness. The cluster level is defined by police stations. Twelve stations will be allocated to the intervention condition and 12 to a wait list control condition. This design was chosen because intervention activities to improve working conditions will be conducted at the workplace level, reflecting normal practice within the host organisation, thus precluding random assignment of individual participants to intervention or control within a given police station. The mental health literacy intervention is also directed at the station level, which is anticipated to affect knowledge, attitudes and helping behaviours across each participating station.

Due to resource limitations, particularly the small number of project field staff available for recruitment, data collection, and intervention implementation, the trial will be rolled out in four temporally-staggered strata of six stations each (sets of six randomly assigned to three intervention and three control stations): a staggered cluster design. A refined version of the intervention will be implemented in control stations by Victoria Police following the conclusion of the trial.

Ethics approval was granted by Human Ethics Advisory Group at the School of Population and Global Health at the University of Melbourne (ethics number: 1340429) and the Human Ethics Committee at Deakin University (Melbourne, Australia) (ethics number: 2014-132), as well as approval from Victoria Police. The study will be conducted in accordance with CONSORT guidelines [45].

### Setting and participants

Victoria Police is a large state-wide organisation comprising ~13,000 uniformed or ‘sworn’ police members and ~3500 civilian employees, operating an estimated 346 police stations across the state. Victoria has a population of 5.9 million persons and covers an area of 227,416 km^2^.

#### Recruitment of clusters

**Fig. 1:**
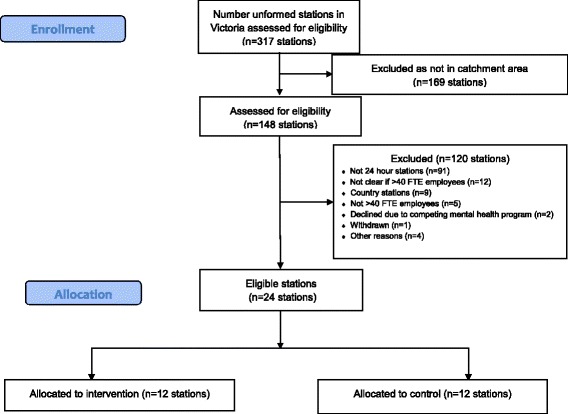
Inner and outer Eastern and Northwest police districts (compass direction relative to the Melbourne central business district) chosen to obtain variation in setting and population base

For feasibility reasons, recruitment will be restricted to the divisions of Victoria Police located in geographical regions that are within two hours’ travel time by car from the researchers’ university base; this corresponds to a large proportion of the state’s population. These divisions were selected in consultation with welfare-related departments within Victoria Police (e.g., Policy Psychology, OH&S, HR) and a steering group consisting of Assistant Commissioners, Commanders, and other senior members of Victoria Police. The inner and outer Eastern and Northwest police districts (compass direction relative to the Melbourne central business district) were chosen to obtain some variation in setting and population base (Fig. 1). The selected areas include a mix of predominantly suburban stations, with some regional sites also involved. In addition to being in the selected areas, station inclusion/eligibility criteria include a minimum size (40 or more sworn police members working in each station to achieve a participating sample of at least 20 employees) and 24-hour operation. The two stations that participated in the intervention development study preceding the trial were excluded from consideration. We recruited police stations in four strata, each consisting of six police stations (three allocated to intervention and three allocated to wait-list control). The Assistant Commissioners for the two participating districts (one per district) generated lists of stations meeting the inclusion criteria. Researchers then made contact with those stations and invited them to take part in the study.

Station-level organisational data will be obtained from Victoria Police’s central HR department for nominated stations, including size (number of sworn members assigned to station), station-level unplanned absence rates, and the proportion of junior to senior members in the station (ratio of employed Full-time Equivalents of constables and senior constables to Sergeants and Senior Sergeants). These station characteristics will be considered in randomisation procedures as described below.

#### Recruitment of participants within stations

All sworn Victoria Police members within participating police stations will be invited to take part in assessments and intervention activities, including early career officers (Probationary Constables and Constables), mid-tier ranks (Senior Constables and Leading Senior Constables) and station command (Sergeants and Senior Sergeants). There are no exclusion criteria at the individual level.

Information regarding the project activities will be distributed in hard-copy leaflet and poster format in participating stations. Information sessions will also be held for station command and general staff, explaining the goals of the study, outlining what the baseline survey involves, and emphasising that participation in the survey is voluntary and anonymous. They will also be informed that their station may be randomly-assigned to receive the intervention, or that they may receive an adapted version of the intervention from Victoria Police at the conclusion of the study (wait-list control). The introduction to the questionnaire will reiterate the voluntary and anonymous nature of the survey and hence completion of the questionnaire will signify that informed consent has been gained.

### Sequence generation, randomisation and allocation concealment

Randomisation to either the intervention or control arms of the trial will be at the station level. A computer-generated randomisation list stratified by region (Inner Eastern, Outer Eastern, Inner North West, and Outer North West) will be created by a trial statistician who is not an investigator in the study. Given the small number of clusters involved, there is a risk that the randomisation process would produce an imbalance between the two treatment groups; to prevent this from occurring, the randomisation procedure will be balanced by size of police stations, mean unplanned absence rate at the station level in the six months prior to the trial, and the proportion of junior to senior members in each station [45, 46]. Study sites will be enrolled and then randomised until the required number of clusters and sample size is reached. As mentioned above, we will recruit in four strata. Within each stratum, there will be three stations allocated to intervention and three allocated to wait-list control. The sequence will be generated through a computer generated list of random numbers using Stata for Windows, version 12 (StataCorp, TX, USA) with an allocation ratio of 1:1. Participating police stations will be informed of their allocation to intervention or control conditions by researchers after completion of baseline surveys. Because random allocation will be conducted for each stratum of six stations after completion of baseline surveys, researchers and participants will be blinded to allocation up to the start of the intervention period for each stratum, after which blinding is not feasible due to the psychosocial nature of the intervention.

### Intervention development

We conducted two intervention needs assessment and development projects preceding the cRCT described in this paper. The first was funded by VicHealth (2011-2014) and focused on job stress reduction in Victoria Police. This study included in-depth interviews (*n* = 13) and focus group discussions (*n* = 7) with Victoria Police members spanning a wide range of ranks: from Probationary Constables and Sergeants (station level) to Superintendents (region-wide responsibilities) and Commanders (organisation-wide responsibilities), as well as with OH&S, Police Psychology, and other member welfare departments. The details of this development project are described in a separate report [47]. In brief, the outcomes of relevance to this trial were a strong consensus across ranks on the need for improved supervisory support for junior officers, and a decision to develop and pilot a supportive leadership development and coaching program as a central element of the intervention in the trial. Access to guidance and support from senior members was frequently cited as a source of stress by junior members. High workload was also a salient theme, in particular report-writing, or ‘correspondence,’ was highlighted as a significant burden for junior officers. A specific intervention activity on workload management for junior officers was considered within the context of this trial, but was not included in the end due to resource constraints. As an alternative, workload management was a focus of leadership development and coaching for station command, with the intended outcome being that junior members would receive support and guidance in workload management through supervisory interactions with station command.

The second study was funded by Worksafe Victoria (2012–2013) and involved assessing member mental health literacy needs through additional interviews. A summary of relevant findings are provided below (details reported separately [48]. Five junior (probationary and confirmed and senior constables) and 13 senior (sergeant level and above) uniformed members participated in semi-structured interviews. The findings were combined with previous knowledge and understanding of the Victoria Police work context, including the other development project described above, and incorporated into recommendations for an integrated intervention strategy. Overall, this qualitative information suggested that senior members had better mental health literacy than junior members. Interviewees also identified stigma associated with mental health issues, and further, access to mental health literacy training varied widely across interviewees. Police members who felt they understood how to help someone with mental health problem reported learning these skills through their own life experiences (e.g. in the context of a family relationship), rather than through formal training. Members in leadership roles varied in terms of the degree to which others felt that they could be approached to discuss mental health issues. In addition, members preferred training via case studies, role plays and other mediums, rather than online. Lastly, a number of positive aspects of working at Victoria Police identified, such as meaningful work and camaraderie.

To ensure that our recommendations would be relevant, practical and feasible for Victoria Police, we consulted with seven Victoria Police subject matter experts chosen on the basis of their expertise or experience relating to one or more aspect of the intervention strategy. These included a senior Sergeant with an interest in staff welfare and peer support, a senior OH&S member, a representative from the Police Psychology Unit, two Inspectors with significant staff welfare portfolios, a senior peer support manager, and an Inspector with expertise in Sergeant and Senior Sergeant leadership training. These subject matter experts confirmed and validated the results regarding mental health literacy in Victoria Police, including the suggestions made by members for how to address mental health literacy gaps in this context (e.g., role plays and face-to-face contact rather than online training). They also offered constructive feedback and advice that enabled the intervention strategy to be refined and finalised in preparation for implementation in the trial. In particular, the integrated, station-level approach was seen as novel and promising, as the model drew on existing resources and aimed to deploy these in a more cohesive and sustainable manner. The subject matter experts also endorsed coaching as a way of developing and consolidating supportive management competencies that was compatible with the operational requirements of busy 24-hour stations. Finally, this group emphasised the importance of engaging with station leadership as early as possible and utilising a consultative approach to developing and managing the project.

### Intervention description

**Fig. 2:**
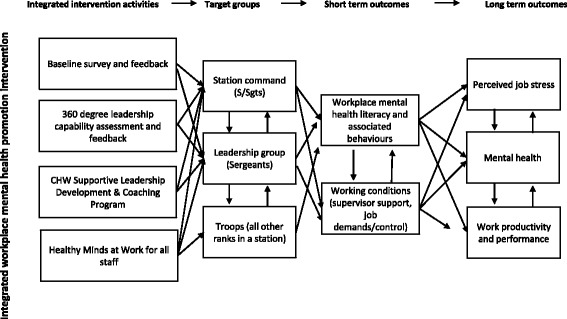
The logic of the intervention--visually relating intervention activities, target groups, proximal outcomes, and secondary or distal outcomes

Following the in-depth intervention development and consultation process outlined above, it was agreed that we would test a station-level intervention aiming to make improvements in two main areas: psychosocial working conditions and mental health literacy. To the extent that improvements are achieved in these two proximal outcome areas, we would also anticipate improvements in the secondary outcomes of member mental health and organisational outcomes. The logic of the intervention--visually relating intervention activities, target groups, proximal outcomes, and secondary or distal outcomes—is presented in Fig. 2. The integrated and mutually-reinforcing nature of the various activities and outcomes should help to facilitate the desired changes in proximal and secondary outcomes. For example, improvements in supportive leadership should reduce job pressures for junior ranks as well as senior ranks (to the extent that they are better able to perform their roles), and parallel improvements in the mental health literacy should facilitate more open, two-way communication and problem-solving around job stress, distress related to factors outside of work, or combinations of the two. Such communication should enable action to identify and control job stressors and/or to seek additional help from internal sources or, where appropriate, health professionals. Furthermore, improvements in working relationships, particularly between junior ranks and supervisors, reinforced by expanded peer support activities, has the potential to improve mental health at all levels of the station hierarchy while also contributing to higher levels of organisational effectiveness.

**Fig. 3:**
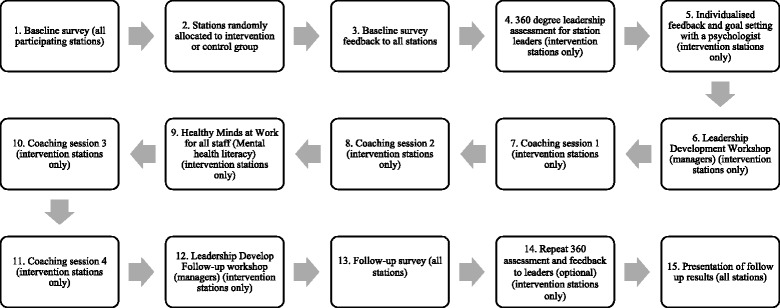
Temporal flow of intervention activities at the station level. Activities include feedback of baseline survey results, a 360 ° leadership assessment for station leaders, the Creating Healthy Workplaces Leading for Wellbeing program for senior sergeants, sergeants and acting Sergeants, Victoria Police “Healthy Minds @ Work” program, peer support program within stations sites

The temporal flow of intervention activities at the station level is presented in Fig. 3. Activities include:

1. *Feedback of baseline survey results*: Members of the research team will present a summary of station-level results to station leaders (those that have a supervisory role, specifically Senior Sergeants, Sergeants and Acting Sergeants) and junior ranking members (those that are below the rank of Sergeant, and do not have a supervisory role), via in-person presentation and posters, respectively. The presentations will be conducted by members of the research team who are degree-qualified at or above a Masters level in Organisation Psychology.
2. *A 360° leadership assessment for station leaders:* This will be followed by a one-hour feedback and development session with a registered psychologist for each participating leader (tailored to their individual 360 assessment): The 360° leadership assessment was developed using the United Kingdom’s Health and Safety Executive’s management competencies for preventing and reducing stress at work, in combination with the Chartered Institute of Personnel and Development management competencies for enhancing employee engagement [49]. To complete the 360° leadership assessment, an individual leader will nominate one manager, five peers and seven direct reports to provide feedback on their leadership behaviours and competencies. Out of the five peers and seven direct reports, the research team will randomly choose three and five, respectively, to maintain respondents’ anonymity. The leader will complete the survey themselves, and their responses will be compared to others’. Once the minimum required number of responses are achieved, the leader will be debriefed by a registered organisational psychologist member of the research team, to explore the links between the individual’s leadership style, station culture and employee wellbeing outcomes, as measured by the baseline survey. Through the 360° debrief process, the leader will be guided to form one strength-based goal (e.g. being personally accessible and using that skill to have more conversations about wellbeing), and one developmental goal (e.g. having difficult conversations with staff in a more productive way).
3. *The Creating Healthy Workplaces Leading for Wellbeing program for leaders (Senior Sergeants, Sergeants and Acting Sergeants):* This program is comprised of an initial five-hour workshop, followed by coaching sessions and concludes with a half-day follow-up workshop. The workshops will be run by members of the research team degree-qualified in psychology and trained in the coaching methodology being used. Workshops are provided to introduce leaders to the competencies that are necessary to ‘lead for wellbeing’, and to provide an opportunity to discuss what specific competencies (e.g., workload management) would look like in the context of law enforcement. The coaching sessions follow the initial one-day workshop and focus on the specific developmental needs of each participating leader as indicated by their 360° feedback. The coaching sessions will be conducted face-to-face, and one-on-one with individual leaders from the stations. Leaders will have a choice of who their coach will be: research team members, external professional coaches, and ‘peer coaches’ (sworn Police members who will be trained in the coaching methodology). Each leader will be offered four one-hour coaching sessions spanning 2–3 months, focusing on skill development specifically related to goals formed during the 360 debrief session.
4. *Victoria Police “Healthy Minds @ Work” program*: This program was developed by registered psychologists within Victoria Police and provides information about common mental health problems such as depression and anxiety, as well as trauma, alcohol use and suicide. Two versions of the program are provided to stations. The first is a one-day leader training session that builds on leaders’ skills to have conversations with their members about such issues, and how to direct them to seek help. Another aim of the program is to decrease stigma around mental health issues and subsequently increase help seeking, both for the leaders themselves and their direct reports. The second is a two-hour mental health literacy session provided to all other ranks in stations. A research team member will attend the two-hour session to explain the relationship between “Healthy Minds @ Work” session and the overall Creating Healthy Workplaces program, so as to highlight that most of the intervention effort is dedicated to improving leaders’ people-management skills and enabling them to better support junior members.
5. *Peer support program within stations sites*: We will make use of an existing peer support program to support and reinforce intervention activities and messages. Peer coaches will be briefed on intervention aims and activities, and will relay this information to peer support officers who operate within stations.

### Wait-list control sites

The same baseline and final assessments will be conducted at control and intervention sites. Victoria Police has committed to delivering a refined version of the intervention (including any changes or refinements indicated by effectiveness evaluation results) to control sites after study results are obtained. There will be no specific project-related intervention activities at control sites during the intervention period. However, control stations will not be prevented from implementing other workplace mental health activities for obvious ethical reasons. Any such activities occurring at control stations should be documented in our process evaluation, as detailed below. To the extent that such activities occur, we will attempt to adjust for them in analyses.

### Data collection

Baseline and follow-up data will be collected at 0 and 12 months respectively using an anonymous online iPad-Mini-based survey. This method was chosen in consultation with Victoria Police, and was informed by a number of considerations. First, identifying information is not usually requested in internal surveys (which average 20–25 % response), and when they do, respondents often leave the field/s blank. It was also anticipated that members would be especially reluctant to include identifying information on a survey that includes questions about mental health. Hence we decided to forgo the collection of identifying information, greatly limiting analysis possibilities, in order to optimise response rates and representativeness in the survey data. It was not possible to use incentives due to Victoria Police anti-corruption policies.

To further address concerns about confidentiality, we will use an electronic survey method that is independent of Victoria Police IT systems. The iPads are clearly marked as Deakin University property, and anonymity and independence from the host organisation’s IT systems will be emphasised in information sessions and promotion activities, alongside information on the voluntary nature of the survey on the welcome page. One or two iPads will be left at each participating station over a period of two to three weeks, during which time the number of responses will be monitored by the researchers on the university server to which the survey data is sent. Response rate updates will be sent to the station ‘champion’ who will help promote survey participation at the stations, and will be complemented by project field staff visits timed to coincide with shift changes to promote the survey.

Individual-level survey data will be complemented by station-level data collected from station command and relevant departments (e.g., HR), and will include staffing levels, changes in key staff at participating stations, restructures, and other events or activities that could affect intervention outcomes. Finally, qualitative interview data will also be collected as detailed below.

### Measures

The survey will contain a range of measures including the following.

#### Socio-demographics

Age group (categorical), sex, highest educational attainment.

#### Employment information

Current job title and rank, time in current position, length of employment in Victoria Police, full- versus part-time.

### Primary outcomes: Psychosocial working conditions and mental health literacy

#### Job control

A 9-item version of Karasek’s measure, including subscales of decision authority and skill discretion [50].

#### Job demands

An 8-item measure of quantitative and cognitive job demands [51].

#### Workplace social support

An 8- item measure including co-worker and supervisor, emotional, instrumental, appraisal and informational support [52].

#### Depression recognition

Will be measured using a written vignette featuring a 30-year-old man named ‘John’ [53, 54]. The vignette covers common symptoms of major depression with suicidal features and meets both Diagnostic and Statistical Manual for Mental Disorders, fourth edition (DSM-IV) and International Classification of Disorders, tenth edition (ICD-10) criteria for this condition [55, 56]. Respondents are asked for an open-ended response to identify what problem, if any, John has.

#### Willingness to assist

The depression vignette is followed by a question asking the respondent if they would help John if he was a co-worker; if yes, the respondent is asked whether the help offered would include any of a list of 6 actions (e.g., suggested they talk to health professional). The respondent is then asked about their degree of confidence in having a conversation with someone at work with a problem like John’s.

#### Personal depression stigma

A measure of stigmatising attitudes towards John as a hypothetical colleague with depression will be assessed using the first six items on the personal subscale of the Depression Stigma Scale [57]. These items assess the degree to which respondents perceived that a person with depression can ‘snap out’ of the problem if they wanted, that depression is a sign of personal weakness, that depression is not a genuine medical illness, and that persons with depression should be avoided as they are contagious, unpredictable, or dangerous.

#### Beliefs about treatment

The vignette is also used as reference to ask the respondent their view on the degree to which various sources of help might helpful or harmful (e.g., seeing their GP, dealing with problem on his own, etc.) [54].

#### Recent helping behaviours

Respondents will be asked to indicate (yes/no/don’t know) whether, over the past six months, they have worked with a colleague suffering from a mental health problem, and if yes, whether they tried to help that person, and if yes, in what ways (six choices, for example: suggesting they see a health professional, plus an open-ended ‘other’) [18].

#### Recent help-seeking behaviours

Respondents are asked “in the past 6 months, if you had a mental health issue, did you seek professional help?,” with response options yes/no/haven’t had an issue. If yes, respondents were asked to indicate the type of help sought (seven choices, e.g., a psychologist, police union, etc.).

#### Help-seeking intentions

Respondents will be asked if they were to experience a mental health issue in the future, how likely they would be to seek help from each of 13 sources (response options on 5-point Likert scale).

#### Help preferences for workplace stress

Respondents will be asked: if they were experiencing workplace stress, how likely they would be to seek help from peers and managers (response options on 5-point Likert scale).

#### Managers’ mental health knowledge, skills and stigma

Respondents at Sergeant rank or higher (only) will be presented with a set of 12 statements on attitudes and behaviours towards employees with mental health problems, gauging their degree of agreement or disagreement on a 6-item Likert-scale [58, 59].

### Secondary outcomes: Mental health & wellbeing, and organisational outcomes

#### Job tension index

A six-item measure of work-related tension and stress [60].

#### Psychological distress

Kessler 6-item measure of psychological distress [61].

#### Job satisfaction

A 14-item measure including subscales of intrinsic and extrinsic job satisfaction [62].

#### Station-level sickness absence rate

Obtained from Victoria Police HR as the average per full-time equivalent (FTE) over six months by station. The validity of this data will be checked by collecting station-reported FTE and comparing this to centrally-provided estimates from HR.

#### Organizational cynicism

A 5-item measure adapted for use in Victoria Police [63] was used to measure employee cynicism towards the willingness and/or capacity of the organisation and its leaders to follow through on promises and to achieve stated goals.

#### Organisational depression disclosure norms

Perceived organisational depression disclosure norms within Victoria Police was measured using four items adapted from Martin et al. [64] (1) *Employees in Victoria Police would be hesitant to disclose that they were suffering from depression*; (2) *In Victoria Police, you should not tell anyone if you have depression*; (3) *Employee depression is considered a suitable topic for discussion in Victoria Police*; (4) *In Victoria Police, your career would be over if you told anyone that you were suffering from depression*. Response options on a 6-point rating scale ranging from ‘1’ (strongly disagree) to ‘6’ (strongly agree).

### Process evaluation

A process evaluation will be conducted to provide narrative insight into the implementation of the intervention. Intervention staff will be asked to record field notes following every intervention activity (noting activity type, where [which cluster], duration, with whom, how many in attendance, etc.) as well as their experiences and impressions following the activity. This will allow for combined collection of quantitative and qualitative process data. Explicit description of the intervention and research process allows for reflexivity among the intervention field staff and will inform data analysis and effectiveness evaluation [65, 66]. Field notes will also be collected following data collection at control stations, with particular attention to workplace mental health-related activities that might occur independently of the project activities.

Semi-structured interviews will also be conducted by a project qualitative researcher with intervention field staff, key project personnel and selected key informants at Victoria Police intervention and control sites as well as some other parts of the organisation (e.g., to identify intervention-independent activities or occurrences that may affect outcomes). The qualitative researcher has extensive experience in conducting interviews and focus group discussions as well as qualitative data analysis.

Focus group discussions will also be conducted with Victoria Police participants at each intervention station after the final intervention workshops. Interviews and focus group discussions will aim to gain insight into (whether and) how the intervention is being, or was, implemented, and how it is being, or was, received at the site/station. The combination of qualitative and quantitative methods outlined for this intervention will enable exploration of pre-existing context-specific factors that may modify intervention effectiveness [67]. This is especially important for this study due to the intervention being implemented in four temporally-staggered sets of 3 stations (each sets of 6 stations is randomised to 3 intervention and 3 control stations) over varying locations. These findings will be used for formative evaluation purposes, referring to the use of these findings to make changes to, or fine tune local intervention activities and strategies.

#### Qualitative analysis

Interviews will be audio taped, transcribed and analysed thematically using *NVivo* for Windows along with intervention staff field notes to describe the evolution of the intervention and what the intervention ‘looked like’ at completion [68]. These analyses will be complemented by the quantitative process data described above to provide a detailed description of intervention implementation across clusters and over time. The quantitative post-intervention survey (described below) will also include questions about participation in specific types of intervention activities to provide measures of intervention reach or penetration (i.e., comparative levels of participation in activities) overall and by cluster.

### Analysis

The following main hypotheses on intervention effectiveness will be tested comparing the intervention versus the control sites at the cluster level based on cluster-wide survey responses:

1. There will be significantly greater improvement in the intervention compared to wait-list control arms in the primary outcomes of mental health literacy and psychosocial working conditions over a one year period;
2. There will be significantly greater improvement in the intervention versus control arms in the secondary outcomes of mental health and organisational outcomes over a one year period;

Analyses will be conducted following CONSORT guidelines for cluster-randomised trials [69]. Because we are not collecting individual identifier information, within-person analyses over time are not possible. A simple two-stage analysis approach will be used: summary measures (e.g., means) will be generated by cluster for pre- and post-intervention, and pre- and post-intervention values will be subtracted to give the mean change by cluster, followed by a two-sample test comparing the summary measures by arm (e.g., t-test, where *n* = total number of clusters). Cluster summaries will be based on census surveys within each station, with all respondents included regardless of whether they participating in intervention activities or not. We will additionally adjust for variation in cluster (station) size by weighting by the number of individuals per cluster. Pending the results of process evaluation, further analyses may be conducted to explore the influence of intervention fidelity and reach, or to attempt to account for activities at control stations that may have influenced outcomes.

### Power and sample size

Sample size was calculated taking into account the cluster design (Table 1). Effect size estimates for mental health literacy outcomes were based on observed significant changes in the primary outcome of mental health knowledge in previous randomised, controlled intervention studies [18, 20]. This is based on a scaled ‘quiz’ score and was the best available measure of workplace mental health literacy. We estimated effect size for changes in psychosocial working conditions based on previous studies [70–72], taking into account distributional methods (which often estimate minimally important score differences as ~0.5 standard deviations [73]) plus the amount of apparent change that is likely attributable to measurement error (based on test-retest correlations over a short period of time [71, 72]).

**Table 1:** Sample size and power calculations for mental health literacy outcomes evaluated in this randomised controlled trial taking into account the cluster design at Alpha = 0.05 and Power = 0.90

	Mean	SD	Effect size (d)	Req’d standard sample size N	ICC	Design effect	Required sample size accounting for cluster design N	Required sample size after allowing for 30 % attrition
Mental health knowledge (*n* = 423)	11.14	3.57	1.79	84	0.050	1.95	164	234
Job control (*n* = 678)	49.82	11.02	8.0	40	0.032	1.60	64	91
Psychological demands (*n* = 700)	14.85	3.73	3.9	20	0.050	1.94	39	56
Colleague social support (*n* = 712)	3.07	1.23	1.2	23	0.048	1.92	44	63
Supervisor social support (*n* = 710)	3.10	1.38	1.4	21	0.019	1.37	29	41

Cluster size was set at 20 based on the rule of thumb for cluster size of m <1/ICC, assuming average ICC ~0.05 (consistent with observed values in Table 1). The ICC was used to calculate the Design Effect sample size multiplier = 1 + (m-1)(ICC). The resulting sample size estimate was then further inflated to allow for 30 % attrition at final. The cluster size of 20 is conservative, representing the minimum size for inclusion. Many clusters/stations will likely exceed this size, thus allowing for less than 100 % recruitment into the study at baseline. Resulting sample size estimates for the intervention arm are presented in the far right column of Table 1. Based on the proxy mental health literacy outcome, we will recruit 12 clusters of >= 20 participants into each arm of the trial (n of 234/20 = 11.7, or 12 clusters per arm).

## Trial status

This trial has been registered with the International Standard Randomised Controlled Trial database (ISRCTN82041334), and the first of four groups of six stations was initiated in September of 2014 has been recruited, baseline surveys conducted, then randomly allocated to intervention and control conditions (3 each); follow-up data for this first stratum is currently being collected. The decision to not collect individual identifying information, along with the administration of surveys on University (clearly not linked in to Victoria Police information systems) iPads likely explains the relatively high baseline survey response rates, averaging approximately 70 % (range 53 to 88 %) across the six stations enrolled in the first stratum.

## Discussion

This project brings together three committed industry partners and internationally leading researchers in the fields of job stress and mental health literacy. A unique strength of this study is the potential to identify preventive synergies that could be realised by improving both working conditions and mental health literacy, meaning benefits in terms of mental health and organisational outcomes that might not be realised by similar improvements in either outcome in the absence of improvement in the other [14]. The intervention strategy was developed in a participatory way in the hope of optimising relevance and feasibility, and thus effectiveness. The joint staffing of personnel conducting the intervention by both the research team and the host organisation (i.e., Victoria Police) is designed to embed capacity development in the project. The program will be refined in light of evaluation results and provided to wait-listed control sites by Victoria Police following the conclusion of the trial.

In addition to advancing this research area internationally, the proposed project will generate evidence-based policy and practice outcomes for the partners involved. The three project partners are well-positioned to disseminate findings and their implications for policy and practice to government, workplace health professionals, relevant non-Government organisations, employer associations, trade unions, and other workplace stakeholders. The approach developed also provides a model of integrated workplace mental health intervention that could be adapted to other workplace settings nationally and internationally.

## Abbreviations

CONSORT: Consolidated Standards of Reporting Trials
cRCT: cluster randomised trial
DSM-IV: Diagnostic and Statistical Manual for Mental Disorders, fourth edition
FTE: full-time equivalent
HR: Human Resources
ICD-10: International Classification of Disorders, tenth edition
ISCRR: Institute for Safety Compensation and Rehabilitation Research
ISRCT: International Standard Randomised Controlled Trial database
MHFA: Mental Health First Aid
OH&S: Occupational Health and Safety
PAR: population attributable risk
